# Magnetization of Ultraviolet-Reduced Graphene Oxide Flakes in Composites Based on Polystyrene

**DOI:** 10.3390/ma14102519

**Published:** 2021-05-12

**Authors:** Alexander N. Ionov, Mikhail P. Volkov, Marianna N. Nikolaeva, Ruslan Y. Smyslov, Alexander N. Bugrov

**Affiliations:** 1 Ioffe Institute, Politekhnicheskaya 26, 194021 St. Petersburg, Russia; ionov@tuch.ioffe.ru (A.N.I.); m.volkov@mail.ioffe.ru (M.P.V.); 2 Institute of Macromolecular Compounds, Russian Academy of Sciences, Bolshoy pr-t 31, 199004 St. Petersburg, Russia; marianna_n@mail.ru (M.N.N.); urs@macro.ru (R.Y.S.); 3 Graduate School of Biomedical Systems and Technology, Institute of Biomedical Systems and Biotechnology, Peter the Great St. Petersburg Polytechnic University (SPbPU), Polytechnicheskaya 29, 195251 St. Petersburg, Russia; 4 Department of Physical Chemistry, Saint Petersburg Electrotechnical University (ETU “LETI”), ul. Professora Popova 5, 197376 St. Petersburg, Russia

**Keywords:** graphene-based materials, ultraviolet-holed graphene nanosheets, polymer-twisted graphene layers, magnetic properties, superconductivity

## Abstract

This work presents our study results of the magnetization of multilayer UV-reduced graphene oxide (UV-rGO), polymer matrix (polystyrene), and a conjugated composite based on them. The mesoscopic structure of the composites synthesized in this work was studied by such methods as X-ray diffraction, SEM, as well as NMR-, IR- and Raman spectroscopy. The magnetization of the composites under investigation and their components was measured using a vibrating-sample magnetometer. It has been shown that the UV-reduction process leads to the formation of many submicron holes distributed inside rGO flakes, which can create edge defects, causing possibly magnetic order in the graphite samples under investigation on the mesoscopic level. This article provides an alternative explanation for the ferromagnetic hysteresis loop in UV-rGO on the base of superconductivity type-II.

## 1. Introduction

As well known, spintronics uses two fundamental properties of the electron: charge and spin. Graphene can be an ideal material for spintronics because of its long charge diffusion length up to room temperature, provided that a ferromagnetic state can be easily created in graphene. Pristine bulk graphite and single or few-layer graphene are nonmagnetic themselves, but the ferromagnetic state occurs in carbon nanomaterials, such as graphene nanoribbons and nanofragments [1].

There have been many theoretical designs of graphene-based structures with magnetism induced by various methods [2]. However, in practical terms, the problem is still far from being solved.

Almost two decades ago, the intrinsic room-temperature ferromagnetic behavior was observed in highly oriented pyrolytic graphite (HOPG) irradiated with high-energy protons [3,4]. Later, this kind of behavior was discovered in graphene after irradiation by ions [5,6]. It was assumed that the controlled introduction of structural defects could make graphene a promising candidate as a magnetic material suitable for spintronics. Irradiation of HOPG with various ions [7,8], including H^+^, C^4+^ and N^4+^ in the mega-electron-volt energy range [6] and helium [3] and iron [6] ones did not show clear indications of long-range magnetic ordering. At the same time, the magnetization data demonstrate ferromagnetic loop behavior up to room temperature. In addition, pure graphite, untreated with high-energy particles, also often has a ferromagnetic type of hysteresis loop [9]. As well known, irradiation with neutrons in graphite can cause numerous defects in it also. Nevertheless, experiments show that only paramagnetism is induced in neutron-irradiated graphite [10,11]. A similar result was obtained upon irradiation with fluorine ions [12].

These contradictory results allow us to conclude that additional studies are needed to clarify the nature of the hysteresis loops in magnetization behavior in bulk and two-dimensional carbon films with macroscopic dimensions. They were inconsistent with the model of point defects in the establishment of long-range ferromagnetic order, with pristine graphite, untreated with high-energy particles, often exhibiting ferromagnetic properties [3,9].

In reduced graphene oxide (rGO), anomalous electrical and magnetic effects can occur due to a change in the conducting π–π* states in the interlayer space [13]. Theoretical calculations previously predicted the magnetic moments, but their origin and relationship with oxygen-containing groups in the layers of graphite oxide and rGO are still questionable. In experimental work, it has been noted that the most nontrivial electrical and magnetic properties are manifested in more defective samples with a significant deviation from stoichiometry. Moreover, they are enhanced by introducing graphene and its derivatives into polymer matrices [14,15]. It turns out that in order to impart magnetic properties to graphene-based composites, it is necessary to abandon the approaches of mixing in the melt [16,17] in favor of obtaining conjugated organic-inorganic materials by copolymerization in situ. Therefore, we pre-modified rGO flakes using 3-(trimethoxysilyl) propyl methacrylate (TMSPM). Another approach was used in work [18]: (*i*) graphene oxide (GO) was grafted by an amino-terminated vinyl polydimethylsiloxane; (*ii*) then, it was covalently bonded with poly(methylmethacrylate) to prepare nanocomposites. Alternatively, the authors of [19] used a different modifier, 3-(aminopropyl)triethoxysilane (APTES), to obtain a covalent rGO conjugate based on poly(vinylidene fluoride) for membrane technology.

This work aimed to study the magnetization of UV-reduced graphene oxide flakes [20] and their composites based on polystyrene (PS) [14,21] with and without weak ferromagnetic behavior. The photoreduction process results in many submicron holes distributed within the UV-rGO flakes, containing edge defective structures comprising epoxy and hydroxyl groups. The effect of ultraviolet irradiation on graphene is similar to irradiation with high-energy protons and, under the model [3,4], should cause a defect-induced magnetic order in the graphite samples under investigation on the mesoscopic level. Indeed, our study showed that the magnetization of UV-rGO flakes has a ferromagnetic type of hysteresis. However, we did not observe an additive behavior of the ferromagnetic magnetizations in the composite where PS showed ferromagnetism. The hysteresis loop for the composite based on ferromagnetic PS was observed to show the same loop as for the single polymer component with ferromagnetism.

Nevertheless, in composites where PS had no ferromagnetism, the composite hysteresis loop was larger than that of UV-rGO flakes. Thus, PS with ferromagnetism suppressed the hysteresis loop of UV-rGO flakes in the composite. This casts doubt on the model of long-range magnetic order caused by point defects in mesoscopic graphite samples with ferromagnetic loop behavior.

## 2. Materials and Methods

Graphite oxide was produced by the modified Hummers method from natural virgin graphite. The technology for obtaining graphene oxide and the analysis of the resulting material were described in detail elsewhere [22]. The reduction of graphene was carried out using ultraviolet irradiation of graphene oxide films [23,24]. The UV irradiation procedure was carried out in an argon atmosphere and described elsewhere [20]. The UV photoreduction of graphene oxide leads to a significant decrease in the total content of the basal plane functional groups, namely epoxy and hydroxyl ones, but with a simultaneous increase in the total number of edge carbonyl groups. However, UV-irradiation is also accompanied by forming many submicron holes in graphene flakes, just as after proton irradiation [3,4]. Figure S1 shows the probable defects in the graphene sheet structure at different stages of obtaining an organic-inorganic composite based on PS-containing submicron particles of graphene nature (in electronic supplementary materials’ file–ESM). Marvin was used for drawing and displaying chemical structures, substructures and reactions [25].

The surface of UV-rGO flakes was functionalized with TMSPM (CAS Number: 2530-85-0, Aldrich, St. Louis, MO, USA), followed by radical polymerization with styrene, forming covalent bonds between UV-rGO and PS chains [14,21]. We used two types of styrene: with and without ferromagnetic ordering. Thus, we had composites with and without ferromagnetic ordering—the composites contained UV-rGO assemblies of several microns’ width and up to 200 nm thickness.

The elemental constituents of composites and components were measured using a TESCAN VEGA 3 SBH scanning electron microscope (TESCAN, Brno, Czech Republic) with an INCA Energy 250/X-max 20 microanalysis system (Oxford Instruments Nanoanalysis Ltd., High Wycombe Bucks, UK).

We have monitored the attachment of the organosilicon modifier to the r-GO surface using FTIR spectroscopy with a Vertex 70 spectrometer (Bruker Optik GmbH, Ettlingen, Germany) equipped with an attenuated total reflectance device (Pike Technologies Inc., Madison, WI, USA).

One has registered Raman spectra of the carbon compounds: non-reduced graphite oxide (GO) and UV-rGO using a 532 nm laser with a power of 0.2 mW in the range of 80–3700 cm^−1^ in a focal field of 25 μm × 1000 μm.

The X-ray diffraction (XRD) analysis of the coherent scattering region (CSR) size and the thickness of graphene layers stacks was performed using a Rigaku SmartLab diffractometer (Rigaku Corporation, Tokyo, Japan) with CuK_α_ radiation.

One has recorded the spectra of UV-rGO treated by organosilicon modifier using the NMR Fourier spectrometer AVANCE II-500WB (Bruker).

The composites under investigation and their components were placed into separate diamagnetic plastic holders with an inner diameter of about 3 mm and installed in a vibrating-sample magnetometer of the PPMS-9 (Quantum Design, San Diego, CA, USA) series. Magnetic measurements on the samples were performed in the temperature range of 5–300 K and with magnetic fields from 0 to ±10 kOe.

## 3. Results

**Figure 1:**
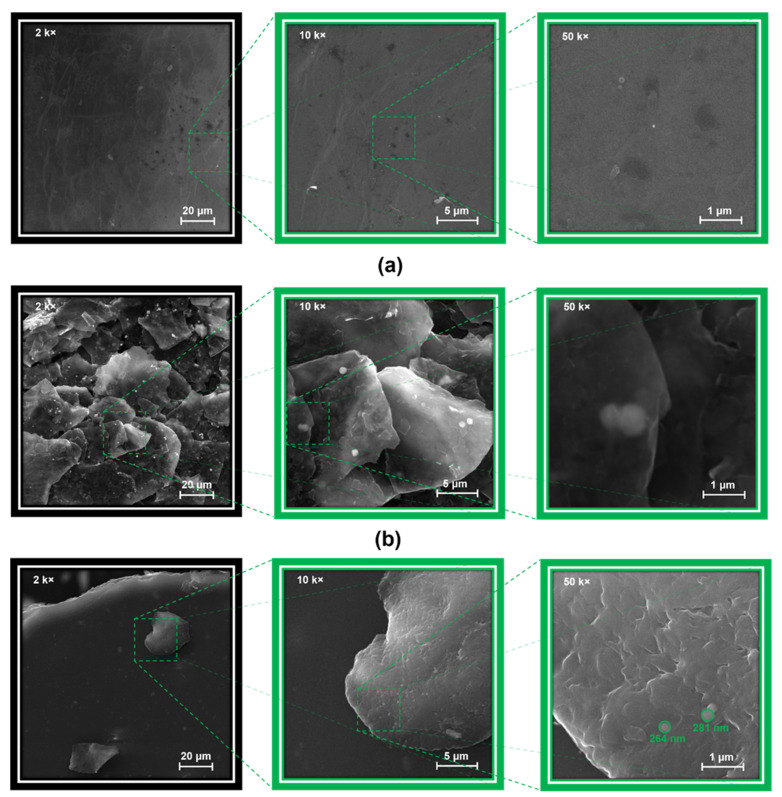
SEM micrographs of the initial GO (**a**), surface modified UV-rGO flakes (**b**) and a film surface for PS/UV-rGO@TMSPM composite (**c**) taken at 2 k×, 10 k× and 50 k× magnifications.

According to SEM micrographs, submicron three-dimensional UV-rGO structures obtained by photoreduction in an argon atmosphere are multilayer films of hundreds of microns in length and width, containing quasi-spherical defects (Figure 1). The diameter of holes formed into graphene sheets using UV radiation lies in the range of 200–800 nm (Figure 1a). As a result of surface functionalization of the UV-rGO macrofilms by vinyl groups, they are crushed under ultrasonic treatment into smaller fragments several tens of microns in size (Figure 1b). Further copolymerization of UV-rGO flakes treated by TMSPM with styrene does not lead to a significant change in their dimensions (Figure 1c).

Elemental analysis of the PS/UV-rGO@TMSPM composite and its components using energy-dispersive X-ray spectroscopy showed only carbon, oxygen, silicon and sulfur in them. After irradiation of GO with ultraviolet light, the amount of oxygen in it significantly decreased, which indirectly confirms its partial photoreduction. Silicon atoms observed in UV-rGO flakes functionalized with TMSPM may indicate the attachment of an organosilicon compound to the defects on the graphene surface (Figure S1). Since the process of GO synthesis includes the treatment of pristine graphite with concentrated H_2_SO_4_ as an intercalant [22,26], then during its subsequent transformation into the oxidized form and washing, sulfur-containing groups may remain in the sample [27]. According to the results of EDX analysis, the composite itself does not contain anything except oxygen and carbon, resulting from the too-low content of TMSPM in it (Table 1).

The structure and thickness of the UV-rGO flakes were determined using X-ray diffraction and Raman spectroscopy. According to X-ray diffraction data [28], the thermal reduction of GO in air occurs above 200 °C, manifesting itself in the disappearance of the reflex at the 2θ angle maximum of 10.5°. This reflex characterizes the increased distance between graphene sheets due to the presence of oxygen-containing functional groups (–COC–, C=O, –OH, COOH) both in the basal plane and at the edges.

**Figure 2:**
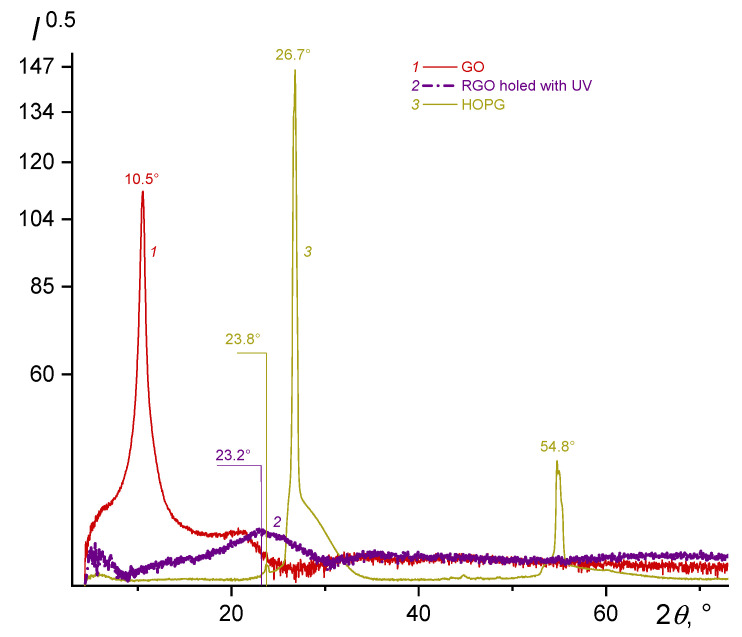
XRD patterns for graphite oxide GO (1), UV-rGO (2) and HOPG (3).

In our case, when studying graphene holed by UV radiation, in the X-ray diffractogram near 2θ at ca. 24°, a wide reflex appears, corresponding to the graphite plane (002) (Figure 2, curve 2). During the reduction process, most of the oxygenated functional groups are removed from the surface of graphene oxide sheets, shortening the distance between the layers. According to Bragg’s law, this circumstance leads to a shift of the reflex to the region of larger angles. It was shown that upon reduction of GO in ultraviolet light, the number of graphene sheets in a stack decreases from 9 to 2 (Table 2). Thus, the UV-rGO flakes are characterized by the absence of galleries consisting of many graphene layers (Figure 2, curve 2). The diffractogram (Figure 2, curve 3) of highly oriented pyrolytic graphite (HOPG) is shown as a reference. The characteristic narrow reflection at 26.7° in the diffractogram indicates many graphene layers in the HOPG galleries. The observed shift to larger angles from 10.5 to 26.7° is due to the closer alignment of graphene planes to each other as compared to graphite oxide (curve 3).

**Figure 3:**
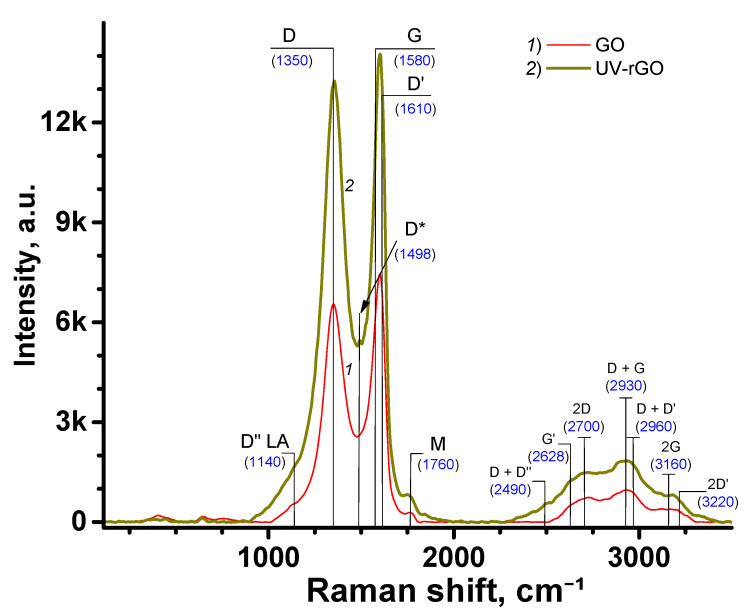
Raman spectra for GO (1), UV-rGO (2).

Figure 3 compares the Raman scattering (RS) spectra of GO with UV-rGO (Table 1). The deconvolution of the Raman curves obtained was carried out according to model concepts [29,30] using the Voigt function in the OriginPro 2021 (version 9.8.0.200) [31] software (Figure S2). The Raman spectral profiles for the carbon nanostructures under study are approximately the same. Furthermore, in the spectral profiles, one can deconvolute the D* peak at 1498 cm^−1^, related to amorphous carbon phases [32]. The complex band of 2300–3300 cm^−1^ may be associated with the sum of the D and G modes and some others (See Table S1 in ESM).

For non-reduced GO, the galleries’ thickness was estimated from the intensity ratio at the maxima of D and G’s vibrational mode bands. This thickness can be interpreted as the size of the cluster diameter or in-plane correlation length, $L_{a}$, following Tuinstra and Koenig [33]:

(1) $$\frac{I_{D}}{I_{G}}=\frac{C\left(\lambda \right)}{L_{a}}$$

where *C*(*λ*) is ca. 4.4 nm, and *λ* is an excitation laser wavelength (514 nm). The $L_{a}$ magnitude for GO is found to be approximately 5.0 nm. Thus, when considering the XRD data obtained (Table 2), the value of $L_{a}$ is 1.5 times smaller than the coherent scattering domain given by the Scherrer formula (7.9 nm).

**Figure 4:**
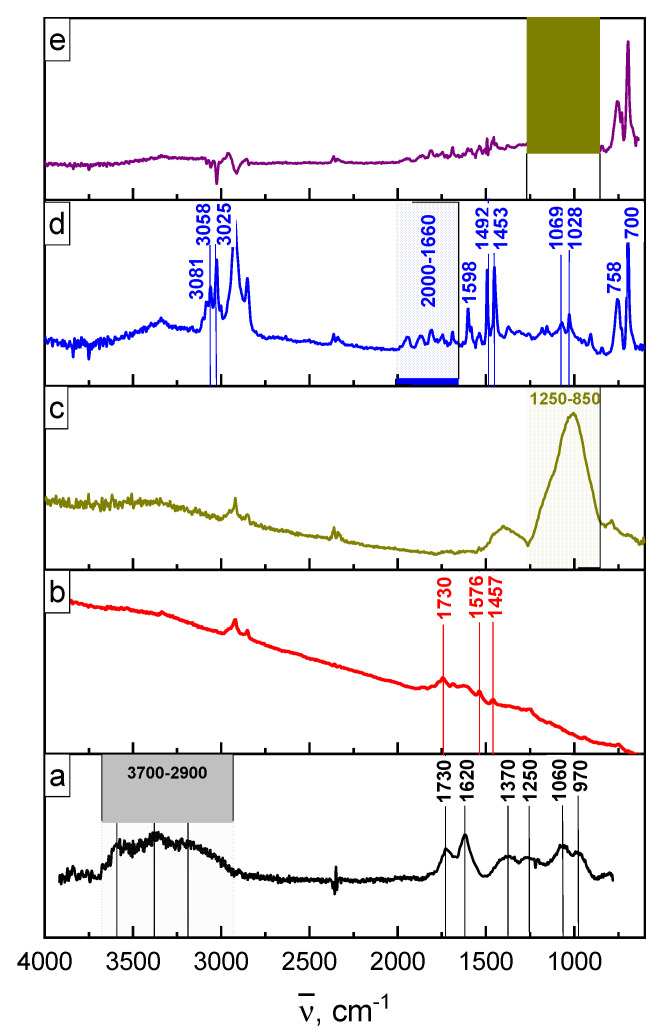
FTIR spectra of graphite oxide (**a**), UV-rGO flakes (**b**), UV-rGO flakes modified with TMSPM (**c**), PS/UV-rGO@TMSPM composite (**d**) and the difference between composite and PS matrix (**e**).

Comparison of FTIR spectra of the initial and UV-exposed graphite oxide indicates its partial reduction (Figure 4). Non-reduced GO is characterized by a broad absorption band in the region of 3700–2900 cm^−1^ with maxima at around 3590, 3404 and 3190 cm^−1^ (Figure 4a). In work [34], they were attributed to the stretching vibrations of the associated and free OH groups, due to adsorbed and coordinated water molecules, respectively. The band at 1730 cm^−1^ indicates stretching vibrations C=O of carboxyl and carbonyl groups, according to [35]. The OH bending vibration of water molecules and C=O skeleton one of GO aromatic rings are observed near 1620 cm^−1^ [35,36]. The absorption bands observed for our graphene oxide with maxima at 1370 and 1250 cm^−1^ are referred in the literature to skeletal vibrations of C–OH, C–O–C bonds [37]. The remaining signals in the short-wavelength region (1060, 970 cm^−1^) can be attributed to edge phenols, epoxy and ether groups [34]. After photoreduction of GO under the action of UV radiation, its FTIR spectrum changes dramatically. The amount of adsorbed moisture and OH groups is significantly reduced (Figure 4b). In addition to the already existing bands at 1730, 1620, 1250 cm^−1^, signals of C–C stretching vibrations (1457 cm^−1^) and C=C stretching ones of the sp^2^ hybridized carbon atoms (1576 cm^−1^) appear in the spectrum [28,38].

The attachment of an organosilicon modifier to the surface of partially reduced graphene oxide is recorded by the presence of absorption bands in the wavelength range of 1250–850 cm^−1^ (Figure 4c), which correspond to stretching vibrations of Si–O, Si–OH, Si–O–C and Si–O–Si bonds [19,39]. Since the UV-rGO flakes functionalized with CH_2_=CH– groups were introduced into PS in small amounts (3 wt.%), their absorption bands are practically invisible against the background of the polymer matrix. In the FTIR spectrum of the PS/UV-rGO@TMSPM composite (Figure 4d), bands were identified that correspond to the vibrations of the C=C bonds of aromatic rings at 1598, 1492 and 1453 cm^−1^ [40]. The peaks in the range of 2000–1660 cm^−1^ can be attributed to monosubstituted benzene rings. The rest of the bands belong to the stretching and torsional vibrations of the C–H bonds (3081, 3058, 3025, 1069, 1028, 758, 700 cm^−1^) [41]. The difference FTIR spectrum of the composite and the PS matrix (Figure 4e) demonstrates only the presence of weak signals in the region of the TMSPM absorption bands [14].

**Figure 5:**
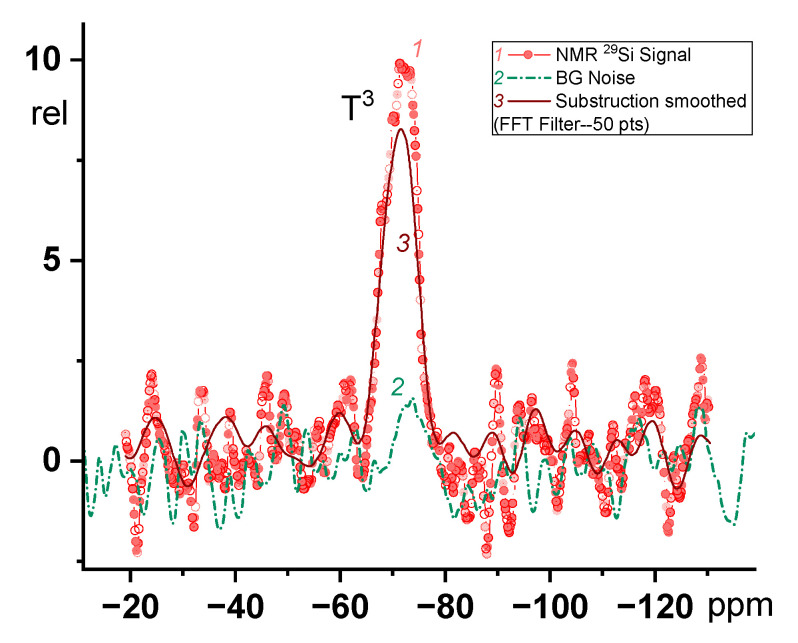
^29^Si NMR-spectra of UV-rGO modified with TMSPM (1), background noise (2) and (3) subtracted by (2) on (1), smoothed using 50 points by FFT filter in the OriginPro 2021.

The nature of the attachment of TMSPM to the UV-rGO surface can be judged from the data of ^29^Si NMR solid-state spectroscopy. For registering the spectrum, a sample functionalized with vinyl groups was mixed with dielectric nanoparticles in a ratio of 1:30. As shown in Figure 5, the ^29^Si NMR spectrum in the entire range of chemical shifts contains a single peak in the region overlapping −65 to –70 ppm for the T^3^ signal corresponding a silicone structure (SiO)_3_Si*(CH_3_) [42,43]. In this paper one can deal with the assumed silicon structure (≥C_edge_–O–)_3_Si*(CH_3_) corresponding to forming covalent bonds between the TMSPM molecule and the edge defects (epoxy and hydroxyl groups) of the UV-rGO holes (Figure 5). This assumption may explain the observed FWHM range of −67 to −75 ppm for this peak (curve 3) and its mismatch with the above T^3^ range. However, there is a match when TMSPM was attached to ZrO_2_ nanoparticles [44]. Because of the absence of peaks at 100 ppm (e.g., (SiO)_3_Si*(OH), signal T^OH^ = Q^3^) and additional contributions in the ranges −45 to −50 [(SiO)Si*(OH)_2_(CH_3_), signal M^(OH)2^] and −55 to −60 ppm [(SiO)_2_Si*(OH)(CH_3_), signal D^OH^], it can be concluded that self-condensation of TMSPM molecules with each other does not occur [42,43,45].

**Figure 6:**
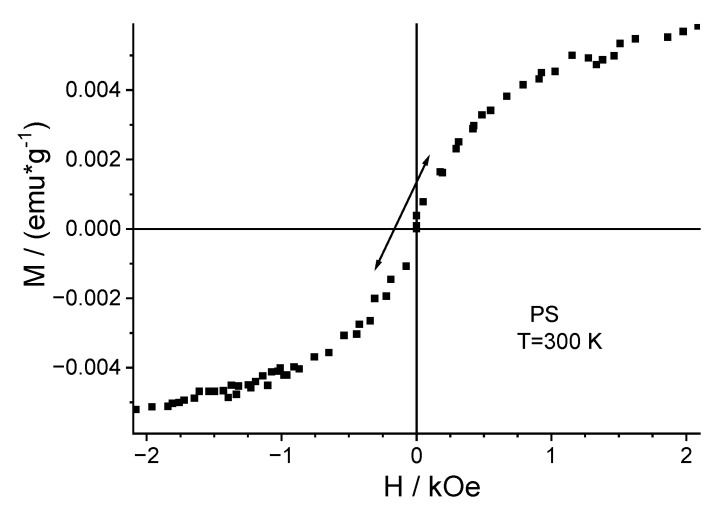
Magnetic field dependence of the specific magnetic moment for the PS at *T* = 300 K. This dependence is typical of the entire temperature range under study.

Figure 6 shows the dependence of the magnetization on the magnetic field for PS, typical for the temperature range of 5–300 K, after subtracting the corresponding diamagnetic component. Polystyrene has magnetization, which hardly depends on the temperature without a hysteresis loop, and typical for paramagnets. It should be noted that the magnetic moment of the sample holder is small and independent of temperature [46].

**Figure 7:**
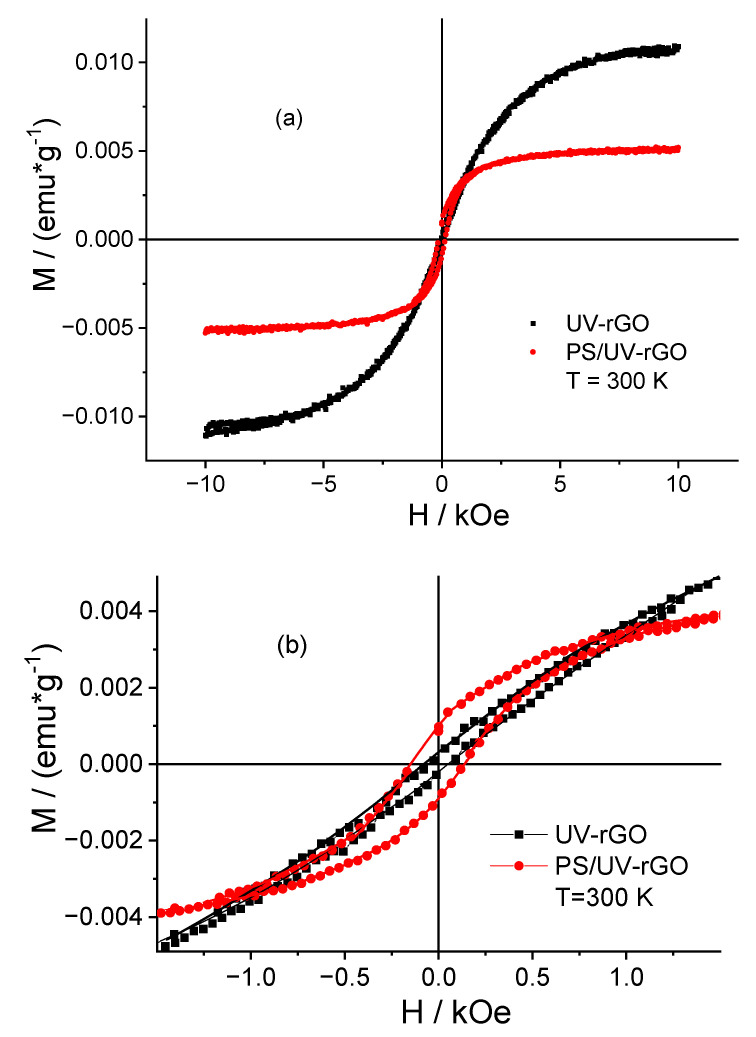
Magnetic field dependences of the specific magnetic moment for UV-rGO-flakes (black curve) and their composite (red curve) based on nonmagnetic PS at various scales (**a**,**b**).

Figure 7 shows the dependences of the magnetic moment of UV-rGO flakes and their composite based on paramagnetic PS on the magnetic field on various scales at a temperature of 300 K after subtracting the corresponding diamagnetic components (see part a). UV-rGO flakes have a magnetization with a hysteresis loop, which at first sight could be explained by ferromagnetism due to the formation of many submicron holes in UV-rGO flakes, as after proton irradiation (Figure 7b) [3,4].

However, in our case, the hysteresis loop is hardly associated with ferromagnetic ordering. Indeed, in a composite based on PS with paramagnetic properties, the hysteresis loop is noticeably more prominent than in UV-rGO (cp. loops in Figure 7b). Within the ferromagnetism model, this means that the ferromagnetism of the composite based on nonmagnetic PS is much greater than the UV-rGO ferromagnetism. The only possible reason for the increase of ferromagnetism in the composite may be due to the appearance of deformation stresses in UV-rGO flakes owing its covalent bonds with PS.

**Figure 8:**
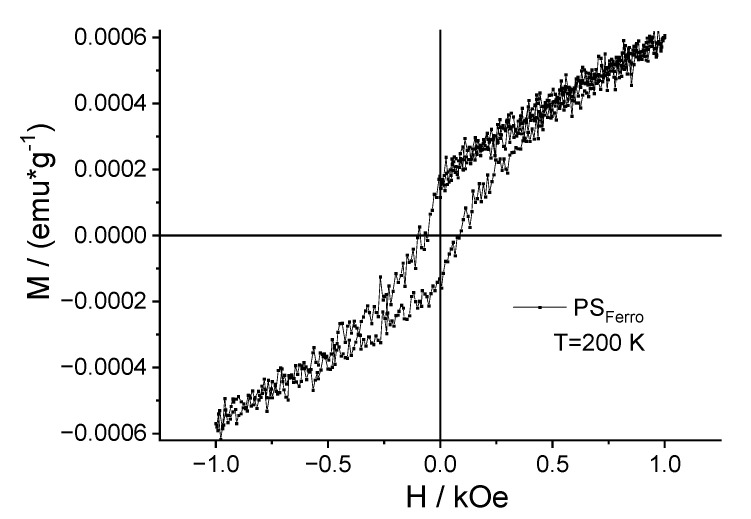
Magnetic field dependence of the specific magnetic moment for the PS_Ferro_ at *T* = 200 K. This dependence is typical of the entire temperature range under study (5–300 K).

In that case, one can expect that the introduction of a small concentration of magnetic impurities into PS can only slightly increase the size of the hysteresis loop but in no way decrease it. To test this hypothesis, we performed a comparative analysis of magnetization behavior depending on the magnetic field between UV-rGO samples with the largest hysteresis loop of the ferromagnetic type and a composite based on the polystyrene doped with a small concentration of ferromagnetic impurities (PS_Ferro_). Figure 8 shows the field dependence of the static magnetization for the PS_Ferro_ at *T* = 200 K after subtracting the corresponding diamagnetic component. 

According to the dependence *M*(H) obtained, polystyrene exhibits ferromagnetic behavior characteristic of the entire investigated temperature range (5–300 K).

**Figure 9:**
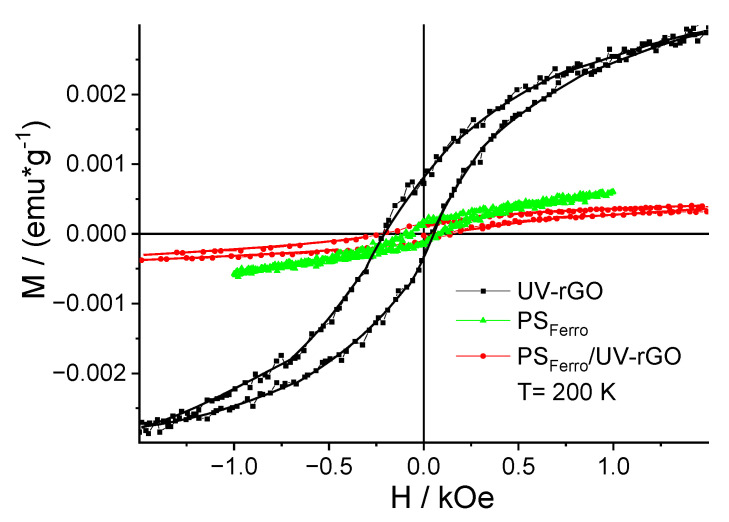
Magnetic field dependences of the specific magnetic moment for UV-rGO flakes (black curve), PS_Ferro_ (green curve) and a composite (red curve) based on magnetic PS_Ferro_ at *T* = 200 K.

Figure 9 shows the dependences of the magnetic moment of UV-rGO flakes, a composite based on ferromagnetic polystyrene and ferromagnetic polystyrene on the magnetic field at a temperature of 200 K after subtracting the corresponding diamagnetic components. As can be seen from Figure 9, the PS_Ferro_/UV-rGO composite has a hysteresis loop of the same size as PS_Ferro_ and much smaller than UV-rGO. Hence, it follows that the introduction of a magnetic impurity into PS suppresses the hysteresis magnetization loop in UV-rGO due to the appearance of an internal magnetic field inside the composite. This effect is observed in type-II superconductors only [47].

In graphene, the valence and conducting band structure are conical, i.e., the density of states (DOS) at the Fermi level is zero. In this case, following the classical Bardeen–Cooper–Schrieffer theory of superconductivity, where the critical temperature T_c_ depends exponentially on the DOS at the Fermi level, T_c_ should be zero. Therefore, to obtain superconductivity at room temperature, it is necessary to change the DOS radically.

According to some theoretical models, T_c_ is predicted to range up to room temperature if the DOS is reconstructed, forming so-called flat bands in graphene [48,49,50,51]. It is supposed that such a DOS reconstruction will occur if graphene, multilayer graphene or carbon flakes are subjected to mechanical stress [52,53,54,55]. As mentioned in the introduction, the photoreduction process results in forming numerous submicron holes within the UV-rGO flakes. Structural defects can lead to strong internal mechanical stresses, which transform the DOS to the flat bands with T_c_ up to room temperature according to the theoretical scenarios described above. Radical polymerization with styrene and formation of covalent bonds between UV-rGO and PS chains should increase the mechanical stresses in UV-rGO flakes. It was shown in [14] that the combination of carbon flakes with paramagnetic magnetization and superconductivity could lead to the illusion of ferromagnetic ordering.

## 4. Conclusions

To conclude, we note that hysteresis observed in UV-rGO flakes at high temperatures can be caused exclusively by superconductivity rather than by ferromagnetic ordering. Indeed, our study showed that the magnetization of UV-rGO flakes has a ferromagnetic type of hysteresis. Furthermore, the hysteresis loop of UV-rGO flakes was much larger than that of PS, having additives with weak ferromagnetic behavior. However, we did not observe an additive behavior of the ferromagnetic magnetizations in the composite based on PS_Ferro_ and UV-rGO flakes. The hysteresis loop for the latter was observed to be of similar dimensions for a polymeric matrix with ferromagnetic additives. Thus, the polymeric matrix having the ferromagnetic behavior suppressed the hysteresis loop of UV-rGO flakes in the composite. Nevertheless, in composites where PS had no ferromagnetic additives, the hysteresis loop was larger than that of UV-rGO flakes. Indeed, this magnetization behavior for the composite can hardly be explained by magnetic ordering.

The data obtained in this work on the study of the dependence of the magnetization on the magnetic field in UV-rGO and composites based on it with PS is in complete agreement with the previously obtained results [14,46].

## Figures and Tables

**Table 1:** EDX analysis data of graphene oxide components for organic-inorganic composites.

System	Elemental Composition at. %			
	C	O	Si	S
GO	62.95	36.79	–	0.26
UV-rGO	74.21	25.47	–	0.32
UV-rGO modified by TMSPM	74.59	24.78	0.28	0.35
PS with UV-rGO modified by TMSPM	96.03	3.97	–	–

**Table 2:** Calculation of CSR sizes according to X-ray diffraction analysis data for GO and UV-rGO using the Scherrer method ^(1)^.

Symbol ^(2)^	Sample	2*θ* ^(3)^, °	FWHM ^(4)^, °	*D_p_*^(5)^, HM	*d*^(6)^, HM	*n* ^(7)^
GO	Graphite oxide	10.5	1.06	7.86	0.84	9
UV-rGO	UV-reduced GO	23.5	9.9	0.86	0.38	2

Note: ^(1)^ Scherrer’s constant is 0.9; irradiation was performed with the Kα^1^-line of copper with a wavelength *λ* = 0.15418 nm; ^(2)^ symbol for a carbon filler of a polymer matrix in a composite; ^(3)^ doubled Bragg diffraction angle; ^(4)^ full width at half height of the reflex at 2*θ*; ^(5)^; *D_p_*—average CSR size according to the Scherrer formula; ^(6)^ *d*—the distance between adjacent crystallographic planes; ^(7)^ *n*—number of graphene layers in a multilayer graphene stack.

## Data Availability

Data is contained within the article or supplementary material. In addition, the data presented are available on request from the corresponding author.

## Supplementary Materials

The following are available online at https://www.mdpi.com/article/10.3390/ma14102519/s1, Figure S1: The schematic diagram for the graphene oxide, UV-reduced graphene oxide sheets and composite UV-rGO/polystyrene. Marvin was used for drawing and displaying chemical structures, substructures and reactions, MarvinSketch 21.3.0, ChemAxon (https://www.chemaxon.com, accessed on 10 May 2021), Figure S2: Deconvolution of the Raman spectrum for the GO obtained. Table S1: The position of different modes in cm^−1^ in the Raman spectrum for GO. See Figure S2.
