# Supplementary material for: Magnetization of Ultraviolet-Reduced Graphene Oxide Flakes in Composites Based on Polystyrene
Source: Materials (Basel). 2021 May 12;14(10):2519. doi: 10.3390/ma14102519 (PMC8151044; doi:10.3390/ma14102519)
Supplement: Supplementary file 1 [file materials-14-02519-s001.zip › materials-1187805-supplementary.pdf]

materials

Supplementary Materials

Magnetization of Ultraviolet-Reduced Graphene Oxide Flakes in Composites Based on Polystyrene

Alexander N. Ionov [1], Mikhail P. Volkov [1], Marianna N. Nikolaeva [2], Ruslan Y. Smyslov [2,3] and Alexander N. Bugrov [2,4,*]

[1] Ioffe Institute, Politekhnicheskaya 26, 194021 St. Petersburg, Russia; ionov@tuch.ioffe.ru (A.N.I.); m.volkov@mail.ioffe.ru (M.P.V.)
[2] Institute of Macromolecular Compounds, Russian Academy of Sciences, Bolshoy pr-t 31, 199004 St. Petersburg, Russia; marianna_n@mail.ru (M.N.N.); urs@macro.ru (R.Y.S.)
[3] Graduate School of Biomedical Systems and Technology, Institute of Biomedical Systems and Biotechnology, Peter the Great St. Petersburg Polytechnic University (SPbPU), Polytechnicheskaya 29, 195251 St. Petersburg, Russia
[4] Department of Physical Chemistry, Saint Petersburg Electrotechnical University (ETU "LETI"), ul. Professora Popova 5, 197376 St. Petersburg, Russia
* Correspondence: anbugrov@etu.ru; Tel.: +7-812-323-6269

Citation: Ionov, A.N.; Volkov, M.P.; Nikolaeva, M.N.; Smyslov, R.Y.; Bugrov, A.N. Magnetization of Ultraviolet-Reduced Graphene Oxide Flakes in Composites Based on Polystyrene. *Materials* **2021**, *14*, 2519. https://doi.org/10.3390/ma14102519

Academic Editor: Chang-Soo Lee

Received: 1 April 2021
Accepted: 7 May 2021
Published: 12 May 2021

Publisher's Note: MDPI stays neutral with regard to jurisdictional claims in published maps and institutional affiliations.

Copyright: © 2021 by the authors. Licensee MDPI, Basel, Switzerland. This article is an open access article distributed under the terms and conditions of the Creative Commons Attribution (CC BY) license (http://creativecommons.org/licenses/by/4.0/).

1. Materials and Composite Design

Figure S1 shows the probable defects in the graphene sheet structure at different stages of obtaining an organic-inorganic composite based on polystyrene containing submicron particles of graphene nature. Point defects, hydroxyl and epoxy groups, arise during the oxidation of graphite, for example, by the Hummers method from the graphene galleries (1st stage). These point defects are on the surface of exfoliated graphite, which is graphene flakes. The resulting material is graphite oxide composed of graphene oxide flakes. In Figure S1, a graphene oxide sheet is shown, which should be on the surface of the flakes.

In the reduction of graphene oxide, for example, during UV irradiation, some of the oxidized groups are eliminated from its surface (e.g., in the form of CO_2), which leads to the formation of a reduced form of graphene oxide, i.e., rGO (2nd stage). As a result of UV-irradiation reduction, submicron-sized edge defects are formed on the surface. In essence, these are holes in several upper layers of rGO. (Figure S1 shows one layer of rGO, which can be on a flake's surface.) In this stage, edge defects are formed, including oxygen-containing groups such as hydroxyl, epoxy, carbonyl and carboxyl groups (Figure S1).

It is possible to attach an organosilicon modifier containing a vinyl group to these groups (3rd stage). One used 3-(trimethoxysilyl) propyl methacrylate (TMSPM) in this work. Subsequently, it is possible to attach chain polymer molecules with the desired properties through this vinyl group due to *in situ* polymerization (4th stage). Schematically, Figure S1 shows the beginning of the polystyrene chain growth from the TMSPM moiety bound thru the Si atom to an edge defect of the rGO layer. Thus, it is possible to synthesize an organic-inorganic composite based on the polymer, containing fragments of graphene nature.

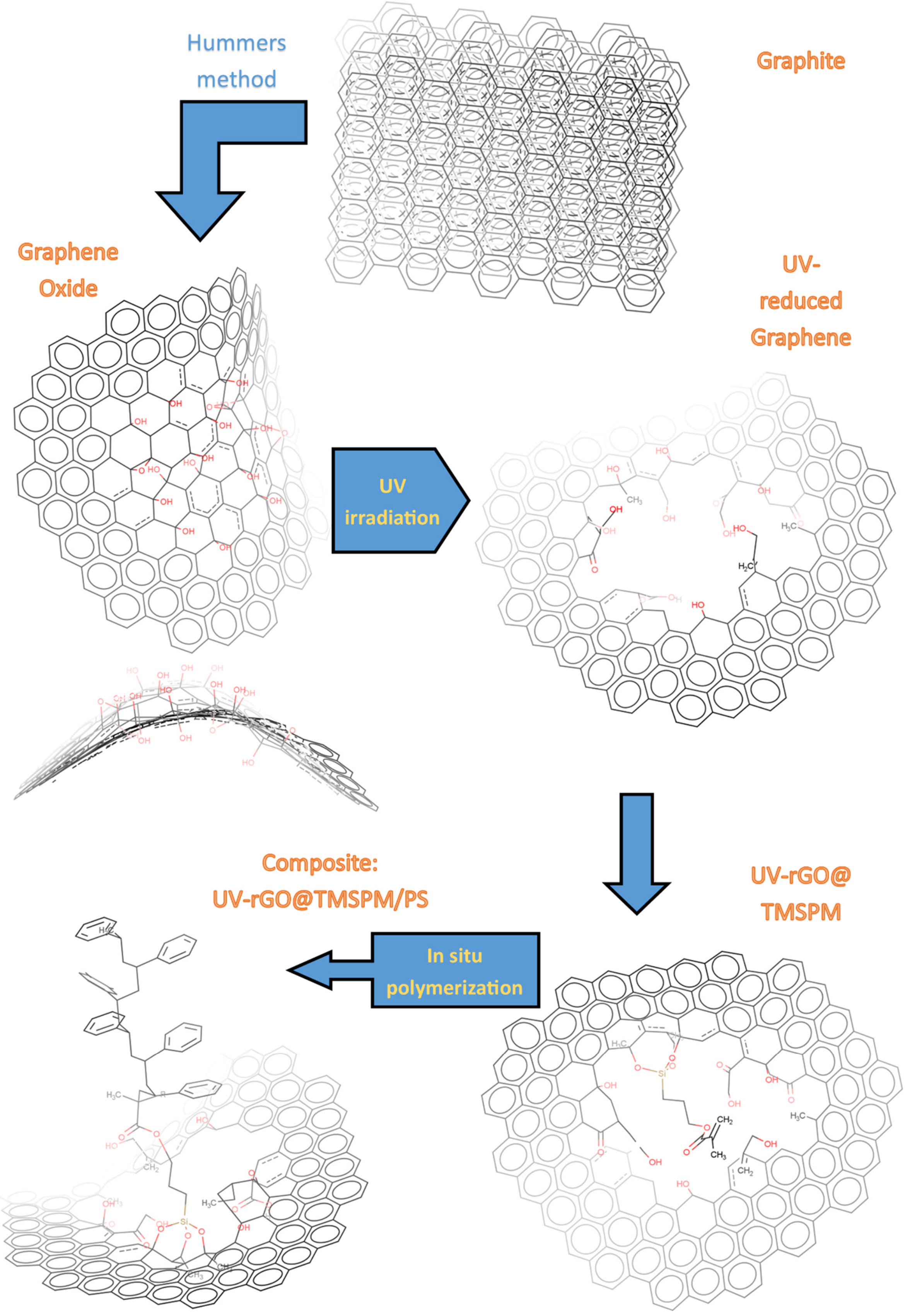

Figure S1. The schematic diagram for the graphene oxide, UV-reduced graphene oxide sheets and composite UV-rGO/polystyrene. Marvin was used for drawing and displaying chemical structures, substructures and reactions, MarvinSketch 21.3.0, ChemAxon (https://www.chemaxon.com).

2. Results

2.1. Raman Analysis of Graphene Oxide

Using model-based notions about the nature of the peaks [1] in the Raman curves, we deconvoluted the spectral contour using the Voigt function in the OriginPro 2021 software. This approach is close to that described in [2]. Figure S2 shows the deconvolution of the spectra for graphene oxide (GO). In these decompositions, only the single parameter y0 was fixed, which corresponds to the subtraction of baseline.

Raman shifts less than 1000 cm^{-1} were not considered since they can be associated with artifacts arising during sample preparation. This range is also challenging to interpret due to the lack of reliable data reviewed in the scientific literature.

Table S1. The position of different modes in cm^{-1} in the Raman spectrum for GO. See Figure. S2.

Mode	Itself	+D″	+D	+G	+D′
D″	1140	-	2490	2753	-
D	1350	2490	2700	2930	2960
D* 1)	1498	-	-	-	-
G	1580	2753	2930	3160	3190
D′	1610	-	2960	3190	3220
M	1760	-	-	-	-

Note: 1) According to Vollebregt et al. [3], the D* band is related to amorphous carbon phases.

In Table S1, we have tried to identify the summed peaks observed in the region of Raman shifts 2300–3300 cm^{-1} for dual modes. The complex band of 2500–3300 cm^{-1} may be associated with the sum of the D and G modes and some others.

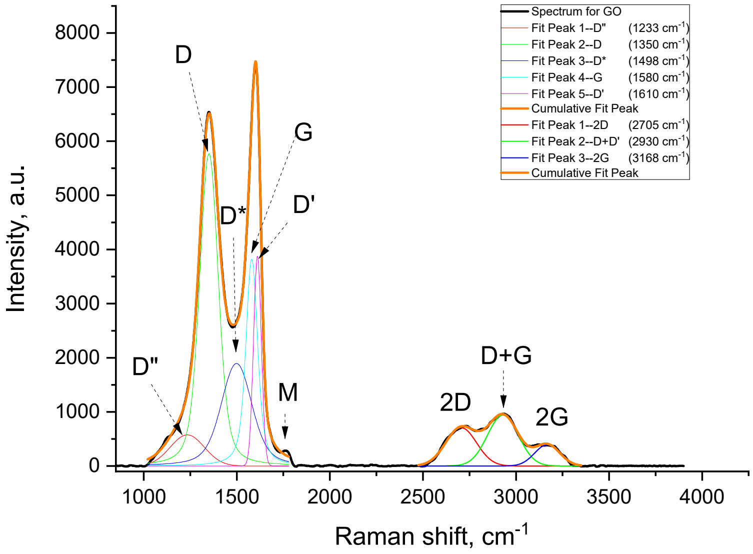

Figure S2. Deconvolution of the Raman spectrum for the GO obtained.

The profiles of the Raman spectra for the carbon nanostructures under study are approximately the same. For non-reduced GO, the thickness of the galleries was estimated from the ratio of the bands corresponding to the maxima of the vibrational modes D and G. It turned out to be about 5.0 nm, which is 1.5 times less than the estimate given by the Scherrer formula (7.9 nm).

References

1. Ferrari, A.C.; Basko, D.M. Raman Spectroscopy as a Versatile Tool for Studying the Properties of Graphene. *Nat. Nanotechnol.* **2013**, *8*, 235–246; doi:10.1038/nnano.2013.46.
2. Ma, B.; Rodriguez, R.D.; Ruban, A.; Pavlov, S.; Sheremet, E. The Correlation between Electrical Conductivity and Second-Order Raman Modes of Laser-Reduced Graphene Oxide. *Phys. Chem. Chem. Phys.* **2019**, *21*, 10125–10134; doi:10.1039/C9CP00093C.
3. Vollebregt, S.; Ishihara, R.; Tichelaar, F.D.; Hou, Y.; Beenakker, C.I.M. Influence of the Growth Temperature on the First and Second-Order Raman Band Ratios and Widths of Carbon Nanotubes and Fibers. *Carbon* **2012**, *50*, 3542–3554; doi:10.1016/j.carbon.2012.03.026.
